# Perforation in an intestinal malignant lymphoma case

**DOI:** 10.1186/s13104-016-2111-6

**Published:** 2016-06-13

**Authors:** Osamu Imataki, Kohei Shiroshita, Shumpei Uchida, Jun-ichiro Kida, Shintaro Akamoto, Makiko Uemura

**Affiliations:** Division of Hematology and Stem Cell Transplantation, Department of Internal Medicine, Faculty of Medicine, Kagawa University, 1750-1 Ikenobe, Miki-cho, Kita-gun, Kagawa, 761-0793 Japan; Postgraduate Medical Training Center, Faculty of Medicine, Kagawa University Hospital, Kagawa, Japan; Department of Gastroenterological Surgery, Faculty of Medicine, Kagawa University, Kagawa, Japan

**Keywords:** Malignant lymphoma, Perforation, Chemotherapy

## Abstract

**Background:**

The gastrointestinal tract is a relatively common involvement site in lymphoma and, in such cases, intestinal perforation is a concern before and during chemotherapy. The prediction of intestinal perforation prior to chemotherapy is difficult, and there is no standard strategy to minimize the frequency of severely adverse gastrointestinal events in lymphoma cases.

**Case presentation:**

The 61-year-old female patient had a history of primary central nervous system lymphoma (PCNSL), diagnosed histologically as diffuse large B cell lymphoma (DLBCL). We administered six courses of intensive chemotherapy consisting of high-dose methotrexate and sequential whole-brain irradiation (40.5 Gy). After a 3-year remission of the PCNSL, the patient’s lymphoma recurred, involving the small intestine. ^18^F-FDG-PET/CT upon the recurrence before chemotherapy showed multiple nodular lesions in the patient’s gastrointestinal tract. Central nervous system lesions were not detected. We administered intensive salvage chemotherapy consisting of cyclophosphamide, high-dose AraC, methyl-prednisolone, etoposide, and rituximab. The response was a rapid partial response, but on day 10 after the initiation of salvage chemotherapy, she complained of abdominal pain with tenderness. The contrast-enhanced (CE)-CT revealed transmural ischemia of the intestine. On the 7th day after the onset of urgent abdominal symptoms, follow-up CE-CT showed that the ischemic lesion had become thin. We conducted elective surgery after waiting for the complete recovery of the patient’s white blood cell count. The pathological findings of resected intestine confirmed the elimination of the majority of lymphoma cells and concomitant partial necrotic tissue.

**Conclusions:**

We were able to avoid the neutropenic period and safely conducted the surgical treatment for the subclinical perforation by using CE-CT. The combination of ^18^F-FDG-PET/CT before chemotherapy and CE-CT scanning for the targeted involvement site helped us evaluate the surgical indications and optimal timing of surgery in a lymphoma patient with gastrointestinal involvement.

## Background

The prevalence of gastrointestinal involvement in lymphoma is relatively common at 22 % [1]. The incidence of intestinal perforation in lymphoma patients in 9 % [2]. In lymphoma patients with gastrointestinal involvement, a primary surgical intervention should be considered before systemic chemotherapy [3, 4] because if chemotherapy will be necessary, neutropenia and other chemotherapy-induced adverse events might hinder the appropriate and optimal surgical intervention [4, 5]. The known risk factors for perforation in gastrointestinal lymphoma patients were discussed in a review [2] which revealed that the small bowel was the most prevalent site of perforation and the diffuse large B-cell disease subtype was the most prevalent lymphoma type that exhibited perforation [2].

Although the appropriate indications and optimal timing of elective surgery have not be determined, it is apparent that emergent surgery at the presentation of perforation should be avoided because of its high morbidity rate. Additionally, a life-threatening condition requiring surgical intervention during chemotherapy is associated with an increased risk of morbidity/mortality [2].

We offer a combinatory imaging evaluation that can be used to estimate the pathological status of gastrointestinal involvement prior to treatment. An exact imaging analysis can determine the degree and severity of involvement sites, and it would contribute to clinical decisions regarding surgical interventions.

## Case presentation

**Fig. 1:**
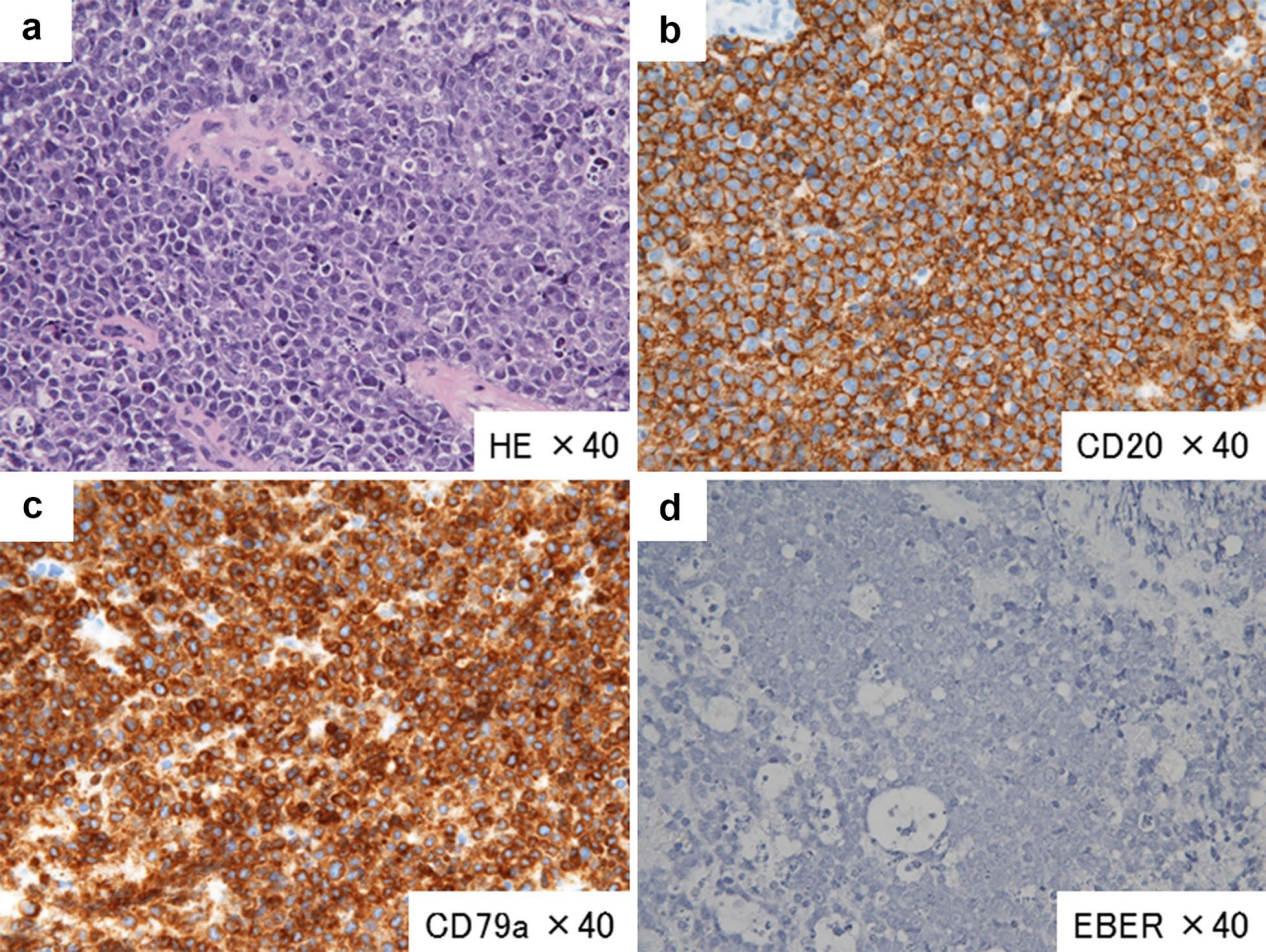
Pathological findings at the onset (brain). Hematoxylin-eosin (HE) staining showed a diffuse infiltration of morphological large lymphocytes (magnification 40×) (**a**). Immunochemical staining revealed positive results for CD20 (**b**) and CD79a (**c**) and a negative result for EBER (**d**)

**Fig. 2:**
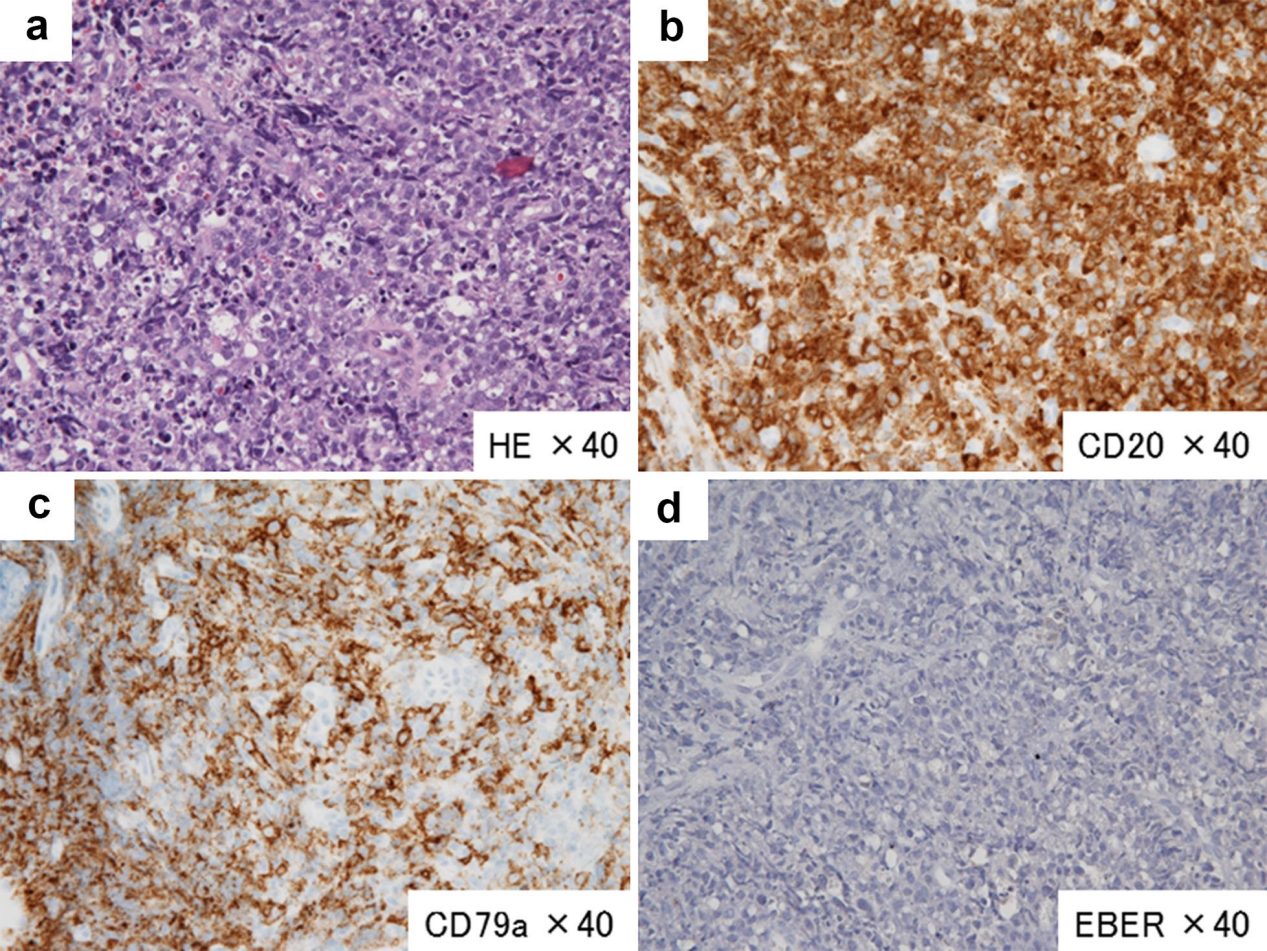
Pathological findings at the recurrence (intestine). HE staining showed a diffuse infiltration of morphological large lymphocytes (magnification as noted) (**a**). Immunochemical staining revealed positive results for CD20 (**b**) and CD79a (**c**) and a negative result for EBER (**d**), phenotypically similar to the primary site, brain (Fig. 1)

**Fig. 3:**
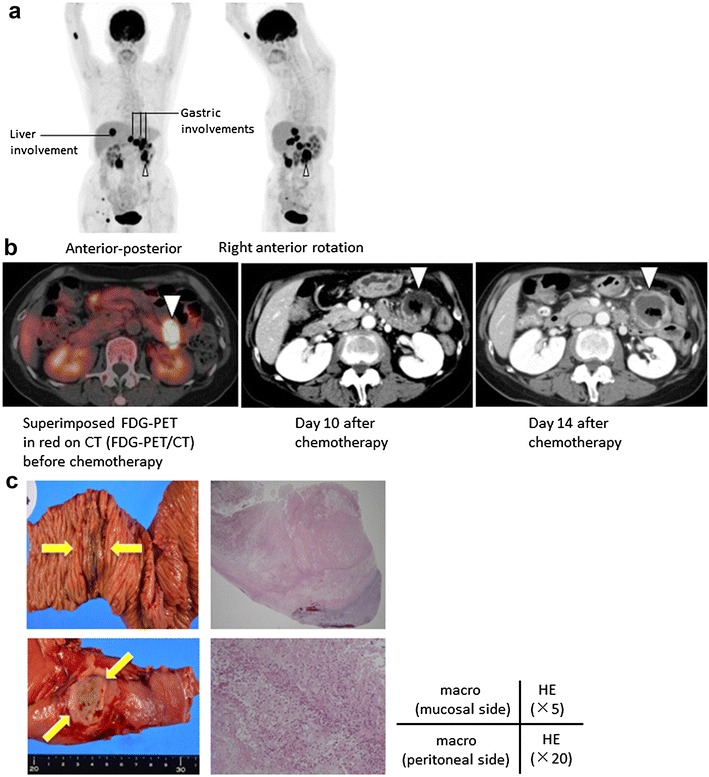
Radiological imaging and pathological findings of the ischemic intestinal site. (**a**) ^18^F-FDG-PET/CT before the patient’s chemotherapy demonstrated the involvement of malignant lymphoma in multiple intestine sites and the liver. The *open triangle* indicates the tissue involvement. (**b**) The ^18^F-FDG-PET image superimposed in *red* on the CT image obtained before the chemotherapy illustrates the transverse image of the involvement (*left*). CT on day 10 after chemotherapy depicting transmural necrosis of the intestine (*middle*). On day 14 after the initiation of the chemotherapy, the enhancement of intestinal wall on CT were recovering (*right*). The *open triangle* indicates the involved intestine. (**c**) Pathological findings of resected intestine confirmed the elimination of the majority of the lymphoma. Macroscopic findings identified the incomplete perforation site covered with a thin serous membrane (*left column*, *yellow arrow*). The pathological findings elucidated the elimination of the majority of lymphoma cells and concomitant partial necrotic tissue (*right column*)

A 61-year-old female had a history of primary central nervous system lymphoma (PCNSL), diagnosed histologically as diffuse large B-cell lymphoma (DLBCL) (Fig. 1). We treated her with four cycles of chemotherapy consisting of cyclophosphamide, doxorubicin, vincristine, prednisolone, and high-dose methotrexate (M-CHOP), and sequential whole-brain irradiation (40.5 Gy). After a 3-year remission of the PCNSL, the patient’s lymphoma recurred as gastrointestinal lesions (Fig. 2). We then performed ^18^F-FDG-PET/CT, which revealed multiple nodular lesions of recurrence in her stomach, small intestine, liver, intraceliac lymph nodes, and bones (Fig. 3a). Cranial MRI detected no aberrant signals as a recurrent lesion of the primary site.

We performed salvage chemotherapy consisting of cyclophosphamide, high-dose AraC, methyl-prednisolone, etoposide, and rituximab (CHASER). The patient then complained of occasional slight abdominal pain after eating, but the symptoms did not disturb her food consumption. She did not need any analgesia for her abdominal pain. The patient was afebrile through until day 9 of this treatment course. On day 10 of the chemotherapy, gastrointestinal perforation was suspected because of abdominal rebound tenderness. Her vital signs on day 10 were as follows: blood pressure 94/56 mmHg, heart rate 68/min, body temperature 36.0 °C. An abdominal X-ray did not reveal free air. An immediate CT scan depicted an enhancement defect of the intestinal wall (Fig. 3b), which indicated transmural ischemia of the intestine. The patient had multiple intestinal involvements of DLBCL, but other infiltration sites of the intestine revealed an enhancement effect of intestinal wall, which had been thought to be intact.

We stopped her oral intake, and we started administering meropenem from day 10. From day 14, spike fever 38.1–39.5 °C was observed, with blood pressure 102/52 mmHg and heart rate 75/min. An elective surgery was conducted on day 18 after the patient’s white blood cell count had risen to >10,000/µL, and we performed a partial resection of the small intestine at 10 cm (anal side) from the Treitz ligament. We used contrast-enhanced (CE)-CT to estimate the surgical indication and optimal timing of surgery. The pathological findings elucidated the elimination of the majority of lymphoma cells and concomitant partial necrotic tissue (Fig. 3c).

## Conclusions

The combination of ^18^F-FDG-PET/CT before chemotherapy and repeated CE-CT scanning for the targeted involvement site was effective for assessing the surgical indication and optimal timing of surgery in this case. The relevant role of ^18^FDG-PET/CT in the delineation of disease-spread patterns has been reported in a study [6] and a review [7]. Although there few studies describing the specific diagnosis and evaluation of small bowel lymphoma, it was reported that ^18^FDG-PET combined with CT is a useful tool for estimating the pathological status of disease [6]. Due to a lack of efficacy of ^18^F-FDG-PET/CT for the evaluation of intestinal perforation caused by cancer, we cannot make conclusions about the utility of this modality. However, ^18^F-FDG-PET/CT is widely applicable for both the localization and viability of malignancies [6]. This property plays a crucial role in assessing disease activities including inflammation and necrosis. We contend that the estimation by FDG-PET before chemotherapy plus repeated CT scanning for the targeted involvement site is the best combinatory technique for making clinical decisions regarding surgical intervention. We should consider this approach for cancer patients with an extra-primary site lesion, in whom necrosis can be critical to the clinical course.

Although intestinal involvement is not a rare complication in lymphoma, clinicians should add a pathological assessment for the etiology of intestinal perforation. Ulcerative bowel lesions may be associated with a particular pathological subtype. Dojcinov et al. [8] reported a series of Epstein Barr virus (EBV)-positive mucocutaneous ulcers associated with various types of immunosuppression. These lesions are histologically characterized by CD30- and Epstein-Barr virus-encoded small RNA (EBER)-positive B cell infiltration, sometimes with Hodgkin-like cells. Dojcinov et al. reported that this type of ulcerative disease exhibits a self-limited indolent clinical course and responds well to conservative therapy, including spontaneous remissions. They proposed that this EBV-positive mucocutaneous ulcer is an independent, newly recognized clinicopathologic entity with Hodgkin-like features and discussed its association with immunosuppression as a common pathogenic mechanism. The localized nature can be managed through treatment. We analyzed the EBER staining in our patient’s primary and recurrence sites and found that both lesions were pathologically EBER-negative. It may be helpful to survey other alternative factors that may contribute to perforation, for the management of intestinal adverse events.

In conclusion, the combination of ^18^F-FDG-PET/CT before chemotherapy and repeated CE-CT scanning for the targeted involvement site can be used to estimate the surgical indications and optimal timing of surgery. We suggest that an elective surgery can rescue lymphoma patients with intestinal involvement [9] under prudent radiological monitoring.

## Abbreviations

DLBCL: diffuse large B-cell lymphoma
EBV: Epstein Barr virus
EBER: Epstein-Barr virus-encoded small RNA
PCNSL: primary central nervous system lymphoma
